# The development of tumour vascular networks

**DOI:** 10.1038/s42003-021-02632-x

**Published:** 2021-09-22

**Authors:** Anahita Fouladzadeh, Mohsen Dorraki, Kay Khine Myo Min, Michaelia P. Cockshell, Emma J. Thompson, Johan W. Verjans, Andrew Allison, Claudine S. Bonder, Derek Abbott

**Affiliations:** 1 Centre for Cancer Biology, University of South Australia and SA Pathology, Adelaide, SA 5000 Australia; 2 South Australian Health and Medical Research Institute (SAHMRI), Adelaide, SA 5000 Australia; 3 Australian Institute for Machine Learning (AIML), The University of Adelaide, Adelaide, SA 5005 Australia; 4 School of Electrical & Electronic Engineering, The University of Adelaide, Adelaide, SA 5005 Australia; 5 Adelaide Medical School, The University of Adelaide, Adelaide, SA 5005 Australia

**Keywords:** Network topology, Tumour angiogenesis, Computational biophysics

## Abstract

The growth of solid tumours relies on an ever-increasing supply of oxygen and nutrients that are delivered via vascular networks. Tumour vasculature includes endothelial cell lined angiogenesis and the less common cancer cell lined vasculogenic mimicry (VM). To study and compare the development of vascular networks formed during angiogenesis and VM (represented here by breast cancer and pancreatic cancer cell lines) a number of in vitro assays were utilised. From live cell imaging, we performed a large-scale automated extraction of network parameters and identified properties not previously reported. We show that for both angiogenesis and VM, the characteristic network path length reduces over time; however, only endothelial cells increase network clustering coefficients thus maintaining small-world network properties as they develop. When compared to angiogenesis, the VM network efficiency is improved by decreasing the number of edges and vertices, and also by increasing edge length. Furthermore, our results demonstrate that angiogenic and VM networks appear to display similar properties to road traffic networks and are also subject to the well-known Braess paradox. This quantitative measurement framework opens up new avenues to potentially evaluate the impact of anti-cancer drugs and anti-vascular therapies.

## Abstract (web summary)

Fouladzadeh, Dorraki and colleagues investigate the development of angiogenic networks for in vitro cancer cell lines. They demonstrate that during the growth stages of vasculogenic mimicry, the number of edges and vertices decreases but the edge length increases resulting in improved network efficiency.

## Introduction

Solid tumours contain robust vascular networks capable of supplying nutrients integral to support cancer progression. Here, we discuss the behaviour of cancer cells over time and gain novel understanding of cancer progression that may provide new knowledge for the development of anti-cancer therapies. The term *network* is a broad description for a set of elements with interactions among them. In biology, there are a number of examples that contain or utilize networks, e.g. protein–protein interacting networks, neural networks, metabolic networks, vascular networks and others. Analysis of networks may potentially address fundamental questions to aid in our understanding of biology. For example, how do gene networks drive the biology of breast cancer^1^? Can network analysis assist C-reactive protein forecasting^2,3^? Which proteins have the highest number of interactions with other proteins? How long does it take for a signal from the brain to reach the limbs? What is the shortest path of reactions that transforms one metabolite into another? What is the role of cell signalling networks in the relationship between molecular species^4^? How does adopting a particular strategy lead to survival in a species under a competition^5^? How does a growing tumour increase its oxygen and nutrient supply? Such questions may potentially be addressed if we investigate the topology and morphology of the networks, i.e., the way contributing factors connect and interact.

In order to analyse biological networks, we mathematically translate them into graphs. In this context, a *network graph* is a mathematical concept containing vertices and edges representing various factors and connections^6^. Therefore, analysing the structure, topology and morphology of the graph representing a biological network may provide useful statistical information, which eventually assists in developing a mathematical or computational model. The challenge with such an approach is that it is not immediately evident how mathematics might elucidate biology. A question motivated by curiosity is: which features are able to distinguish biological networks from other classes of complex physical systems^7^? To address this question, we direct our attention to indicating key parameters for the development of vascular networks within solid tumours over time using graph theory; which bear some similarity to other well-known networks such as road networks, metro maps, power grids, fungi networks, etc. Defining the vascular network of a solid tumour is the overall aim of this study.

Without access to a blood supply, solid tumours cannot grow more than a few millimetres in diameter^8,9^. In order to grow beyond this volume, the tumour initiates a pro-angiogenic switch. Prior studies deem that tumour vascularisation is influenced by a number of parameters such as endothelial cell (EC) migration, proliferation, oxygen availability, the existence of tumour angiogenesis factors and presence of extracellular matrix components^10^. There are various mechanisms for tumour vascularisation including EC sprouting, intussusceptive angiogenesis, recruitment of endothelial progenitor cells, vessel co-option, lymphangiogenesis and vasculogenic mimicry^11^. Here, we consider two types of networks that can contribute to the supply of oxygen and nutrients for cancer progression: (i) angiogenesis (EC lined vasculature) and (ii) vasculogenic mimicry (VM, cancer cell lined vasculature). The vital role of angiogenesis and VM is highlighted in growth, and metastasis of solid tumours^8,12–15^. Briefly, angiogenesis is the process of new blood vessel formation^16^ and involves the growth, migration, and differentiation of ECs. In contrast, VM-competent cancer cells form their own cancer cell-lines channels for blood transport independent of typical modes of angiogenesis^17–19^. In 2016, a meta-analysis was published detailing the 5-year survival outcomes of >3600 patients across 11 different malignancies (including melanoma, breast, ovarian and lung) with results suggesting that VM content within a tumour mass (as identified by lumenised vascular structures that are low/negative for CD31 (or CD34) and stain positive with the periodic-acid Schiff (PAS) reagent) correlated strongly with poor prognosis^20^. Further investigation has identified that VM contributes to tumour vasculature to varying degrees^21^ with one study documenting that in a stage 3 neuroblastoma ~20% of the micro vessels were tumour derived while that of stage 4 rose to 78%^22^. Notably, the literature also indicates that cancer cells and endothelial cells can join forces to form mosaic vascular structures and in vivo imaging demonstrated a physiological perfusion of blood between endothelial-lined vasculature and VM networks (reviewed in ref. ^23^). Taken together, there is growing support to target VM as a novel treatment strategy for the most aggressive and difficult to treat cancers^23,24^. The patterning characteristics of VM, detected by molecular imaging, have proven to be a useful tool to aid clinical practice^24^. Therefore, an analysis based on topologic and morphologic perspectives may potentially be used in VM and angiogenesis distinction and considered as a future tool to assist clinical practice.

In this paper, we develop an understanding of network formation in canonical EC sprouting based on mathematical concepts and graph theory, and address the fundamental question of which mathematical network structure best characterizes angiogenic networks. We also directly compare the development of angiogenic EC networks with cancer cell formed VM networks with a focus on a human breast cancer cell line^25^ (MDA-MB-231 cells and their metastatic derivative MDA-MB-231-LM2) and two human pancreatic cancer cell lines^26^ (BxPC-3 and AsPC-1 cells). This comparison builds on our understanding of the vasculogenic patterns that contribute to cancer progression.

## Results

**Fig. 1:**
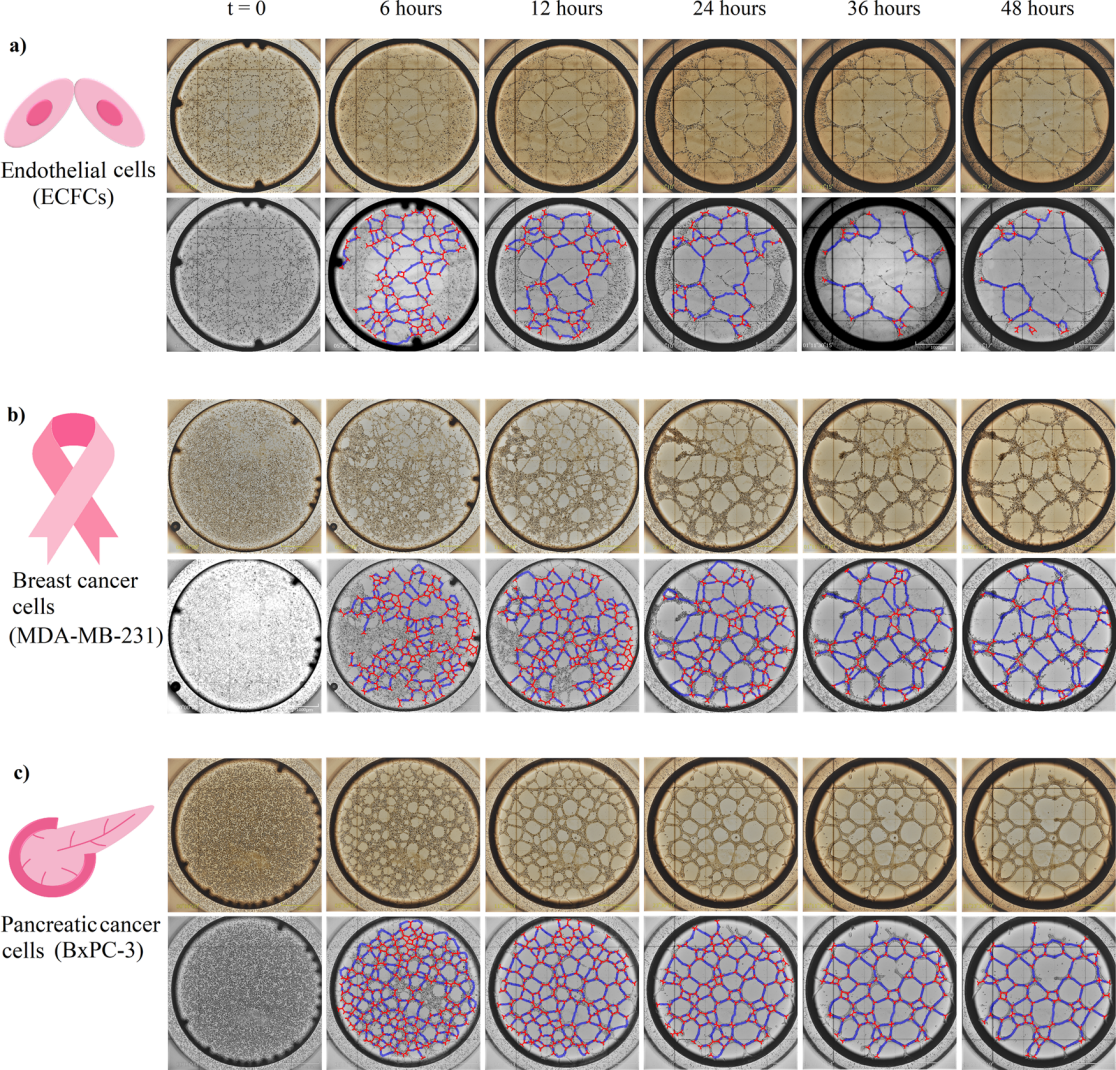
Identifying edges and vertices from in vitro assays showing formation of angiogenic and VM networks. **a** The top panel shows how ECs form an angiogenic network, and how this network evolves over time. In the lower panel, we demonstrate the results from our image processing software that extracted the vertices (red) and edges (blue) from the EC network. **b** Breast cancer cells form a network with short edges in the first 6 h. The length of edges increases and the number of nodes decreases over time. **c** The formation of the network is demonstrated for pancreatic cancer cells. The arrangements of edges and vertices, and also the edge sizes are more evenly spread than in breast cancer.

To investigate the dynamics of this process, we designed an in vitro study to display the networks formed by cells; we investigated the cell growth for angiogenesis using endothelial colony-forming cells (ECFCs) and VM (represented by MDA-MB-231 and BxPC-3 cells). Using real-time microscopy, we captured a number of network images showing the vascular process during the growth phase of these cells (see Supplementary Videos 1–3). Using our customized image processing software, we extracted and displayed the vertices and edges in the networks (Fig. 1).

The results illustrate that the angiogenic networks established by primary ECFCs^27^ from healthy donors possess longer edges in the first 6 h in comparison to those formed by cancer cells; however, in this assay, the stability is relatively short-lived and the network breaks down after 24 h (Fig. 1a). Similar experiments with the MDA-MB-231 breast cancer cell line revealed that a network, containing a large number of edges of short length, forms in the first 6 h. We observed that, compared to the ECs, the number of vertices and edges is reduced after 24 h; but the edge lengths increase significantly, and the network dynamics remain stable at 24 h (Fig. 1b). For the BxPC-3 pancreatic cancer cell line, Fig. 1c shows that the behaviour of these cancer cells is similar to the breast cancer cells with the exception that the networks are almost evenly distributed at all times throughout the investigation.

**Fig. 2:**
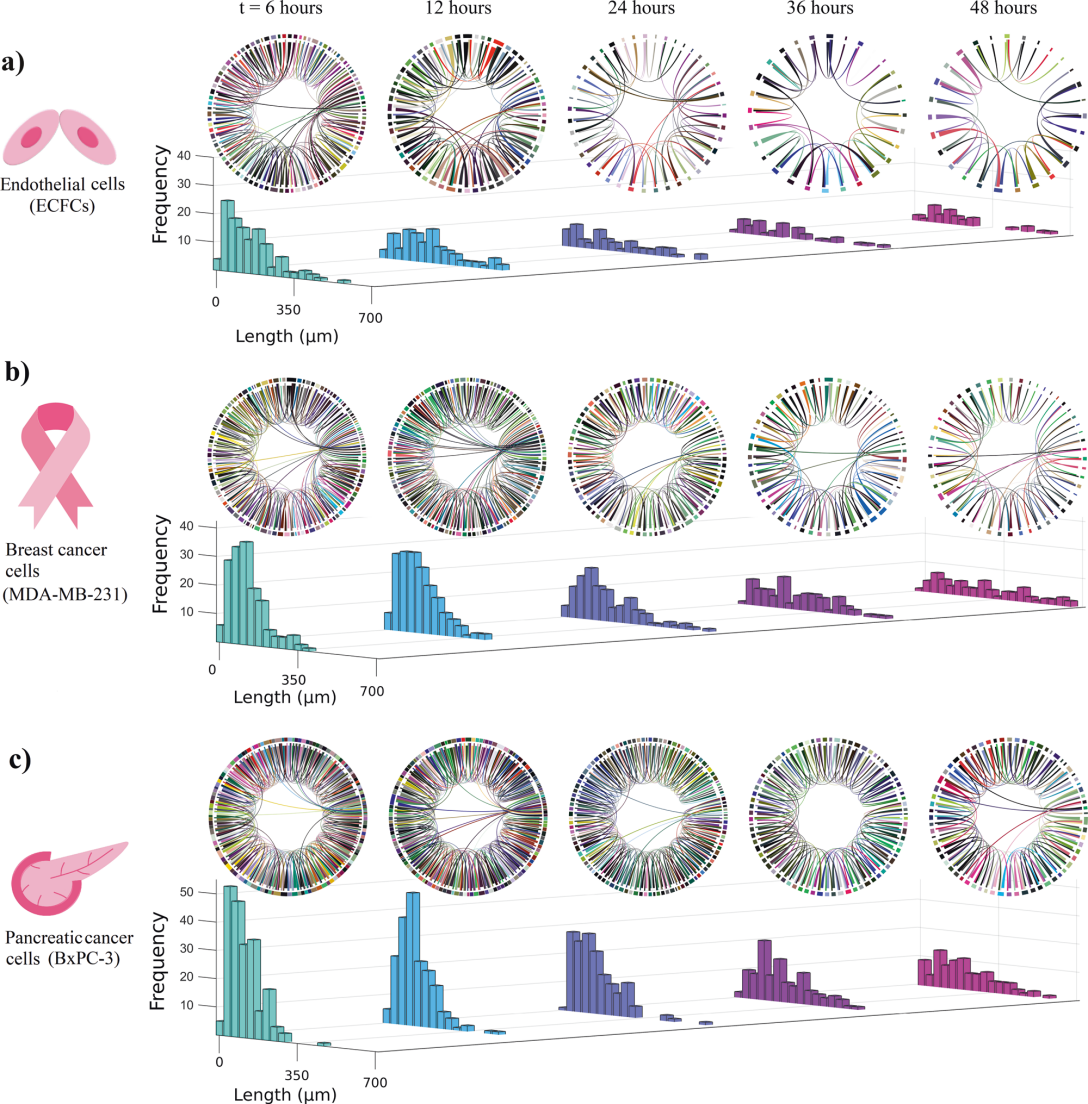
Circular layouts of network growth and distribution of edge lengths. **a** Circular layout of EC network growth determined from the videos of five independent experiments using the EC cells with the average taken over time. The corresponding histograms in the lower panel suggest there are a substantial number of small edges in early stages but the edge lengths increase over time. **b** A similar analysis for the breast cancer cell VM network determined from the videos of five independent experiments. **c** The histograms for pancreatic cancer cell VM network showing the total number of edges over time. Histograms are plotted from five individual pancreatic cancer networks.

To further understand vascular network behaviour, we visualize the networks using circular layouts, in addition, we combine the circular layouts and corresponding histograms are obtained from five individual networks for each cell type showing the distribution of averaged edge lengths (Fig. 2). The circular plot and histogram in Fig. 2a illustrate that the number of vertices and edges in an EC network drops significantly over time, and the network tends to maintain only the long and short edges and not the midrange length edges. Similarly, the MDA-MB-231 breast cancer cells formed a VM network with a large number of short length edges during the early growth stages. The edges then combined together, forming longer edges over time (Fig. 2b). In addition, we observed that the total number of edges and vertices related to the BxPC-3 pancreatic cancer cell VM network is greater than in the breast cancer cell line suggesting greater aggression in formation of the networks (Fig. 2c).

These observations allow us to characterize and compare the vascularisation mechanisms within angiogenesis and VM formation. The results suggest that cancer cells are more stable than ECs (at least in vitro) in their ability to form and maintain vascular networks.

### Are the vascular networks small-world networks?

**Fig. 3:**
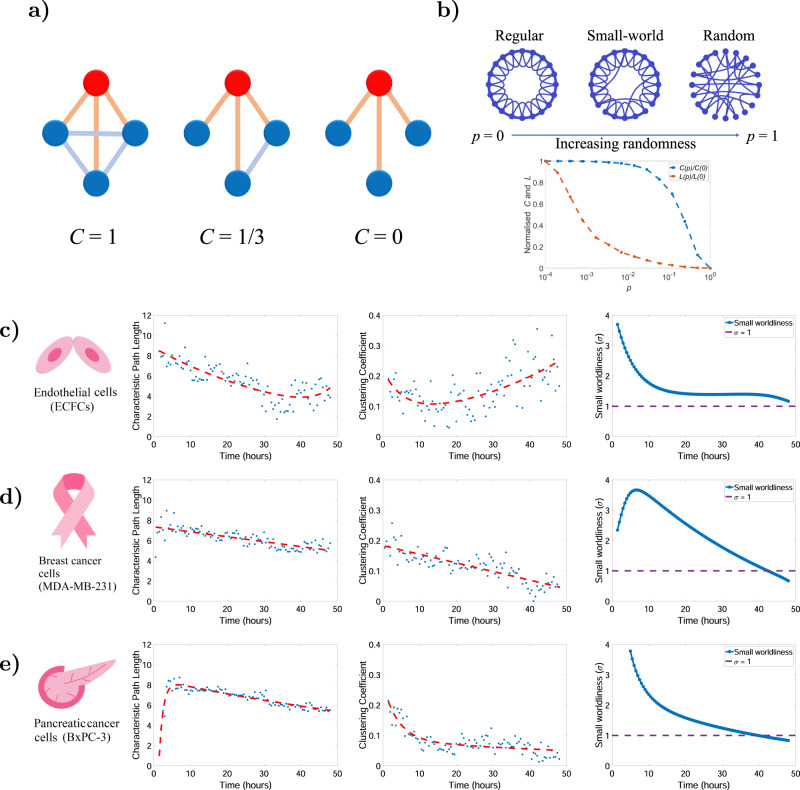
Small-worldness, characteristic path length and clustering coefficient for ECs, breast cancer cells and pancreatic cancer cells. **a** An example of clustering coefficient on an undirected graph is shown. The local clustering coefficient of the red node is obtained as the proportion of connections among its neighbours which are actually realised compared with the number of all possible connections. **b** The Watts-Strogatz model^31^ and the generation of small-world networks. The model starts with a regular lattice network. Then, with probability *p*, edges are rewired uniformly at random such that at $p=0$ the network is a regular lattice and at $p=1$ the network is random. At intermediate values of $p$, the network has so-called small-world characteristics with considerable local clustering (from the lattice network) and short characteristic path length (from the random network). **c** Analysis is of five angiogenic networks formed by ECs with the average shown for each time point. The dashed red line is fitted on the data showing clustering coefficient decays in the EC case; however, clustering coefficient increases. Here, VM networks were similarly analysed from five **d** breast cancer and five **e** pancreatic cancer networks separately and the mean determined over time. Exponential lines are fitted to the characteristic path length and clustering coefficient. Both characteristic path length and average clustering coefficient drop over time in VM networks.

Complex dynamic systems are often characterized by a large number of nonlinearly interacting elements. To investigate this intricate connectivity, we further explored the network clustering coefficients and characteristic path length, which led us to investigate the phenomenon of small-world networks^28,29^. A network possesses a small-world structure when its clustering coefficient (*C*) is relatively high, but its characteristic path length (*L*) is relatively low. This conceptual definition comes from the fact that a high clustering coefficient occurs in the phenomenon of social groups (demonstrated in Fig. 3a), while short mean path length occurs in the phenomenon of rapid information spread^30^. The notion of small-world networks introduced by Watts and Strogatz^31^, illustrated in Fig. 3b, has emerged and has given rise to empirical studies of graphs such as neural networks^32^, biological networks^33^, and transportation networks^34^. There are several indices of small-worldness; here we used the *σ* factor that was initially proposed by Kogut et al.^35^ and widely adopted in the literature. Using an associated statistical test^36^, it can be identified that the values of *σ* *>* 1 are interpreted as evidence of small-worldness (see the ‘Methods’ section).

From the definitions above, we analysed five videos from each cell type, and investigated mean clustering coefficient, characteristic path length and small-worldness for our networks. The decreasing trend in *L* and increasing trend in *C* maintains *σ* above the purple dashed line suggesting that the ECFCs network is small-world in 48 h (Fig. 3c). The number of vertices and edges in the MDA-MB-231 breast cancer cell line network attains its maximum after ~10 h. The decay rate of *C* is greater that *L* in the breast cancer network, and this proportion causes a decrease in the small-worldness factor (Fig. 3d). The maximum number of vertices and edges grows faster for the BxPC-3 pancreatic cancer cell line. The exponential decrease in *C* leads to an exponential decreased trend in *σ* (Fig. 3e). Our observations suggest that although the angiogenesis network formed by ECFCs is less stable than both the cancer cell line VM (MDA-MB-231 and BxPC-3 cells), the neighbours of any given node are likely to be neighbours of each other and most nodes can be reached from every other node by a small number of hops or steps.

### The Braess paradox in the development of networks

After applying our software to five individual videos for each cell type, we further analysed the association of the number of edges and mean edge lengths in the networks formed by ECFCs, MDA-MB-231 and BxPC-3 cancer cells. In addition, we investigated mean edge thickness and length. We observed a network improvement in all three cases, suggesting that although the number of edges decreased, the mean edge lengths increased over time. The edge thickness in breast and pancreatic cancer cells show a smooth pattern while the edge thickness in ECs fluctuates and finally drops significantly.

**Fig. 4:**
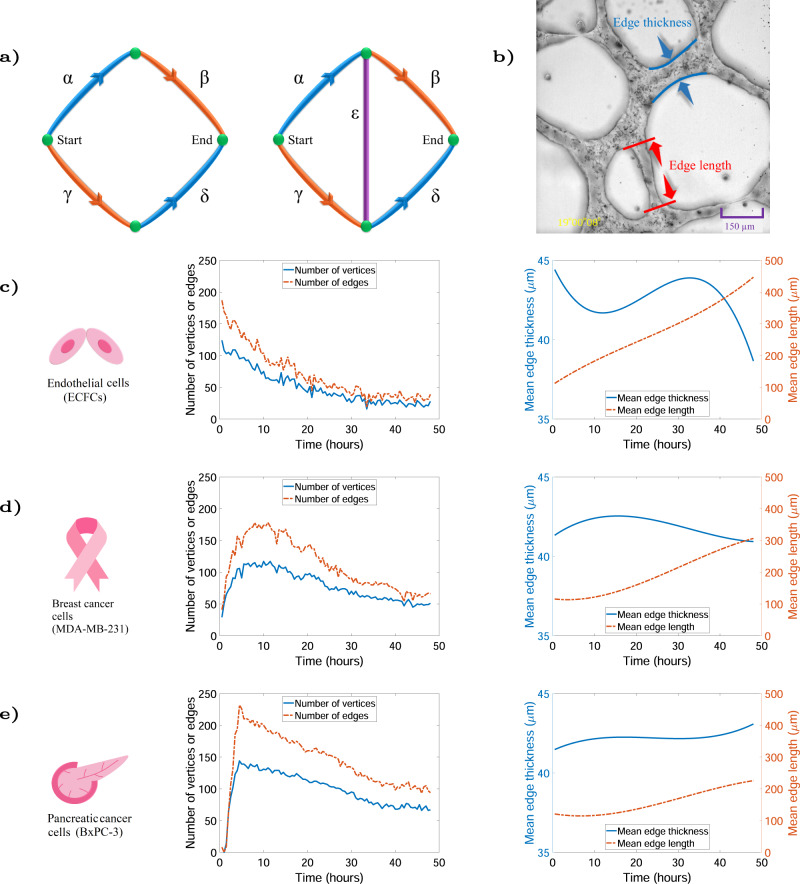
Braess paradox in vascular networks. **a** A traffic network example is demonstrated to explain Braess paradox where a simple network consisting of two routes connecting the start and end points. The travel time along orange roads is given by *ρ*/100 min, where *ρ* represents the number of vehicles, while the traffic time is 45 min for the blue roads. In equilibrium, traffic will distribute evenly between these two routes connecting start to end. Therefore, the travel time for total 4000 vehicles (2000 for each path) is 65 min (45 min for blue and 20 min for orange roads) along each of the two routes. Installation of the purple road with an extremely short travel time of approximately zero minutes on the bottom map offers the third route (*γεβ*). Drivers begin to use the new route, reducing the travel time from 65 to 40; however, as more vehicles choose the new route, the travel time will increase to 80. **b** The edge thickness and length are defined here. **c** The average number of edges and vertices is shown in red and blue lines (left panel), and the mean edge thickness and length are shown in blue and orange lines (right panel) for five EC networks. It may be seen that the mean edge length trend increases significantly for ECs. **d** The graph in part c is plotted for five breast cancer cases. **e** The formation graph in parts (**c**) and (**d**) is plotted for five pancreatic networks. A similar trend can be seen in the rate of mean edge length and thickness in both cancers in part (**d**) and (**e**).

We propose an explanation for this observation by referring to a phenomenon, which is normally associated with road traffic networks, known as the Braess paradox^37,38^. Adding a by-pass road to a traffic network can counterintuitively cause the average travel time to increase—this is known as the Braess paradox (Fig. 4a). The Braess paradox has been explored in other areas such as in electron transport in mesoscopic networks^39^ and in systems of interconnected mechanical springs^40^. Previously, it has been suggested that anti-angiogenic therapy may lead to a normalisation of the vasculature before the vessel network finally collapses^41^ due to the decreasing self-referential connections and increasing vessel stability^42^.

Here for the first time, we have shown that VM by MDA-MB-231 and BxPC-3 cancer cells display network behaviour similar to what is observed as the Braess paradox for traffic networks. We observe that a decrease in the number of edges and vertices in all three cases, as a function of time, leads to an increase in mean edge length showing a trend towards greater network efficiency and avoiding the adverse effects of the Braess paradox (Fig. 4b, c, d). During the early growth stage, the ECFCs reach the maximum number of vertices and edges significantly faster than for MDA-MB-231 and BxPC-3 cancer cells (Fig. 4c). We also notice that the trend in the mean edge length in breast and pancreatic cancer is approximately the same; however, there is a drastic increase in the EC mean edge length. The mean edge thickness in ECs shows a fluctuating pattern and drops significantly during later growth stages.

### Biological similarities between angiogenesis and vasculogenic mimicry

One question that arises from these comparative observations is whether the differences observed in the networks are a consequence of different phenotypic properties between the cells rather than differences in vascular-related events. To address this possibility, we completed additional experiments with the ECFCs and cancer cells (MDA-MB-231 and BxPC-3).

**Fig. 5:**
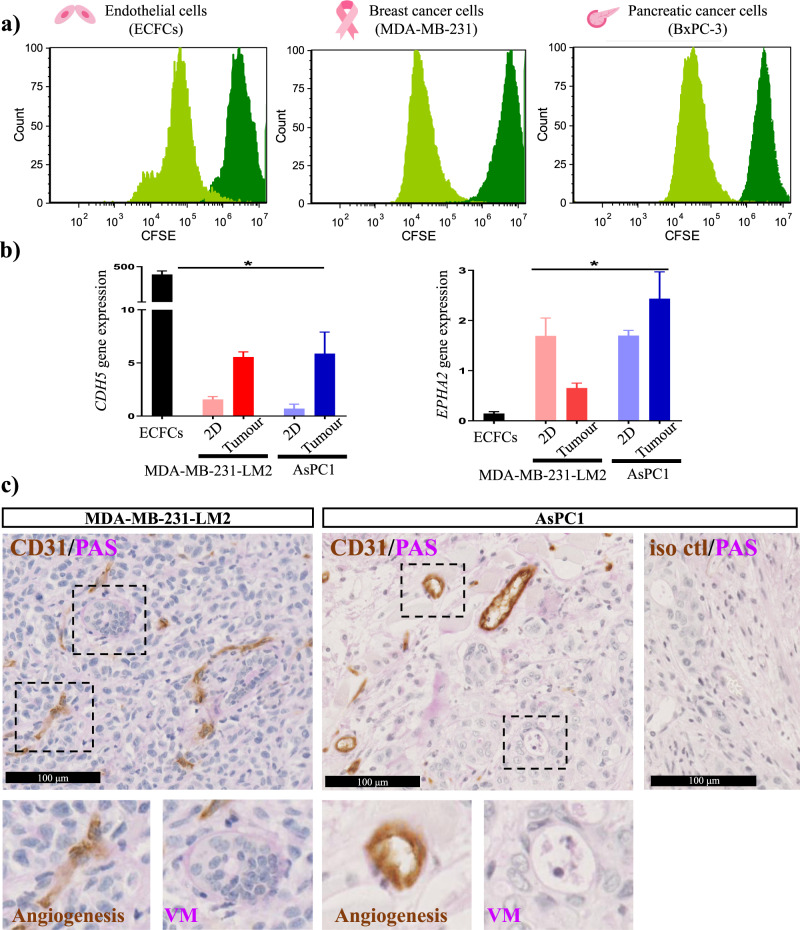
Defining characteristics of angiogenesis and vasculogenic mimicry. **a** Proliferation analysis of human endothelial cells (ECFCs), breast cancer cells (MDA-MB-231) and pancreatic cancer cells (BxPC-3) stained with CFSE and measured by flow cytometry at Day 1 (dark green) and Day 5 (light green). **b** Gene expression profiling of VE-cadherin (*CDH5*) and *EPHA2* in ECFCs grown in 2D tissue culture, MDA-MB-231-LM2 breast cancer cells (2D tissue culture and 3D tumours) and AsPC-1 pancreatic cancer cells (2D tissue culture and 3D tumours). Data are mean + SEM, *n* = 3 biological replicates normalised to the housekeeper gene *CYPA*; *p < 0.05 versus ECs. **c** Histological analysis of 3D tumours (breast cancer and pancreatic cancer xenografts) stained with CD31 (brown) and periodic-acid Schiff (PAS, magenta) counter stained with eosin (scale bar: 100 μm).

First, to compare the proliferation rate of these three different cell types, we included carboxyfluorescein diacetate succinimidyl ester (CFSE) into the cell culture where the fluorescent dye (that stably labels cells) permeated into the cells such that proliferation rate could be determined using flow cytometry^43^. Figure 5a shows an exemplary CFSE histogram of the three cell types with a similar profile of CFSE and demonstrates the similar peaks (dark green). Based on the MFI, distribution of CFSE over 5 days is similar in ECFCs and BxPC-3 pancreatic cancer cells suggesting an equivalent rate of proliferation. Interestingly, the highly aggressive and invasive human MDA-MB-231 breast cancer cells exhibited a slightly faster rate of proliferation.

Next, to investigate and compare the expression of known vascular cell markers we examined the gene expression of VE-cadherin and Ephrin type-A receptor 2 (EphA2) in the ECs and VM-competent cancer cells (breast and pancreatic). Notably, in these experiments, we compared gene expression in 2-dimensional (2D) tissue culture as well as a 3-dimensional (3D) context with tumours harvested from mice and for this we utilized human breast cancer cells (MDA-MB-231-LM2^44^, a VM-competent cell line derived from the metastasis of the parentals MDA-MB-231) and a second VM-competent human pancreatic cancer cell line (AsPC-1)^45^. Figure 5b shows that, ECs express high levels of *CDH5* (VE-cadherin) and that it is also readily detectable in the MDA-MB-231-LM2 breast cancer cells and AsPC-1 pancreatic cancer cells. Notably, when we examined *CDH5* expression in the MDA-MB-231-LM2 and AsPC-1 tumours harvested from mice, we observed a significant increase in expression. In direct contrast, *EPHA2* is lowly expressed in ECs when compared to the 2D tissue culture MDA-MB-231-LM2 and AsPC-1 cells. Interestingly, MDA-MB-231-LM2 tumours harvested from mice exhibit a possible reduction compared to their 2D tissue culture, but still elevated compared to ECs. For the AsPC-1 mouse tumour cells, *EPHA2* appears to be similarly expressed between 2D and 3D.

Finally, histology was performed as further support for the aforementioned cancer cells to undergo VM and form vascular structures in vivo. More specifically, we examined the vasculature of the MDA-MB-231-LM2 and AsPC-1 3D xenograft tumours via immunohistological staining of formalin-fixed paraffin-embedded tumours sections using an antibody to CD31 and PAS stain to define angiogenesis (CD31+PAS+) as well as VM structures (CD31−PAS+). Figure 5c illustrates that within the tumour mass, both angiogenesis and VM structures are detectable.

## Discussion

We have taken theoretical and experimental approaches to study different types of vascular networks. We examined the development of networks formed by ECs (angiogenesis), and directly compared the development to networks formed by cancer cells (vasculogenic mimicry). We have shown that these cells, although derived from different sources, i.e. endothelial cells versus cancer cells, they share common features in the rate of cellular division, expression of vascular proteins and are capable of performing the same acts within a tumour mass, i.e. angiogenesis and VM. Although we focused on topological and morphological phenomena, our scheme for angiogenic network analysis may potentially lead to applicability of network-based approaches for clinical purposes. Our in vitro assays have shown that although angiogenic networks are less aggressive than VM, they maintain small-world properties for a longer time period. Our mathematically modelling of these vascular networks, also revealed that they behave much like automobile traffic on a road network. Utility of this information may be in potentially characterizing how different therapies might lead these networks to starve a tumour from its blood supply. These network-based analyses can potentially be used to develop a variety of cancer models via graph theory. In addition, network-based quantitative measures can open new avenues for evaluating the impact of anti-cancer drugs and anti-angiogenic therapies.

## Methods

### Cell culture and angiogenesis/vasculogenic mimicry assays

Endothelial colony-forming cells (ECFCs (or ECs)) were isolated from healthy human peripheral blood, as previously described^27^, as approved by the human ethics committees of the University of South Australia (UniSA HREC #201187) and SA Pathology (ethics #85–13). Briefly, collagen I-coated plates were seeded with peripheral blood mononuclear cells and cultured in EGM-2 media (Lonza) containing 20% ES cell screened foetal bovine serum (Hyclone, GE Healthcare, Chicago, IL, USA) until colony formation at ~14 days culture after which time the cells were passaged and cultured for no more than nine passages.

MDA-MB-231 breast cancer cells were cultured in Dulbecco’s modified Eagle medium (DMEM, Gibco, Life Technologies, Carlsbad, California, USA) supplemented with 10% FBS (HyClone, Logan, UT, USA) and 2 mM GlutaMax (Gibco) and BxPC-3 pancreatic cancer cells were maintained in RPMI1640 media (Gibco, Thermo Fisher Scientific, Waltham, MA, USA) supplemented with 10% FBS.

For the in vitro vascular assays, 1.5 × 10^4^ ECFCs, 1.5 × 10^4^ MDA-MB-231 breast cancer cells and 3.5 × 10^4^ BxPC-3 pancreatic cancer cells were seeded per well onto 10 µl of Growth Factor Reduced/normal Matrigel (Corning, Corning, NY, USA) in angiogenesis µ-slides (Ibidi, Munich, Germany) for up to 48 h. Time-lapse video of the network formation was taken on the live cell imaging microscope ‘CellVoyager CV1000 Yokogawa Spinning disk Confocal Scanner’ (Olympus Life Science, Tokyo, Japan). Images were extracted from the video post-acquisition.

### The network analysis software

The vascular structures of angiogenesis or VM formation were extracted using our custom MATLAB software. Previous studies normally used manual counting approach for measuring tubular structures in VM. This computational approach, based on image processing tools, assists in extracting useful information from VM networks, avoiding miscalculation. Therefore, we developed a software that receives the network images and precisely outputs a number of useful parameters such as number and position of tubular vessels and junctions, histogram, graph parameters, etc.

The algorithm consists of the following steps:

Step (1) Reading image: Read in the cell images, which are the images of breast cancer cells.

Step (2) Image adjusting: The RGB images are converted to grayscale and image intensity values or colormap is adjusted to improve image contrast. In addition, the area of interest is segmented, and any object out of border of area can be removed.

Step (3) B/W Filtering: The grayscale images converted to B/W, and after image enhancement, the small holes in vessel images can be removed. In addition, the scatter points related to a few cells that are not connected to tubular structures may be removed.

Step (4) Vessel outline extraction: Using this morphological operation, all objects are reduced to lines in 2-D binary images.

Step (5) Finding individual vessel: Tubular junctions are extracted, and the vessels between them identified as an individual vessel. This information assists to calculate the graph parameters.

### Small-worldness, clustering coefficient, and path length

In a network consisting of *N* vertices, the distance *L*_*ij*_ between two vertices, *n*_*i*_ and *n*_*j*_ is given by the length of the shortest path between the vertices, that is, the minimal number of edges that need to be traversed to travel from vertex *n*_*i*_ to *n*_*j*_. The average or characteristic path length *L* *=* *<L*_*ij*_*>* of a network is defined as the average distance between all pairs of vertices^6^. The clustering coefficient relates to the local cohesiveness of a network and measures the probability that two vertices with a common neighbour are connected. In the case of undirected networks, given a vertex *n*_*i*_ with *k*_*i*_ neighbours, there exist *E*_max_ = *k*_*i*_*(k*_*i*_ *−* 1)/2 possible edges between the neighbours. The clustering coefficient *C*_*i*_ of the vertex *n*_*i*_ is then given as the ratio of the actual number of edges *E*_*i*_ between the neighbours to the maximal number *E*_max_, therefore, ${C}_{i}=\frac{2{E}_{i}}{{k_i}({k_i}-1)}$. The small-worldness can be achieved via both characteristic clustering coefficient (*C*) and path length (*L*) with respect to a single reference graph: $\sigma =\frac{C/{C}_{r}}{L/{L}_{r}}$, where ${C}_{r}$ and ${L}_{r}$ are the mean clustering coefficient and characteristic path length for an equivalent random network, respectively^34^.

### Statistical analysis

Data were expressed as mean ± standard error of the mean (SEM). Statistical analyses and significance were calculated by one-way ANOVA Tukey’s multiple comparisons test to determine statistical significance using GraphPad PRISM software (San Diego, CA, USA). In all comparisons, *p* < 0.05 was considered statistically significant.

### Cell proliferation assay

Adherent cells including ECFCs, MDA-MB-231 and BxPC-3 were labelled using a Vybrant™ Cell Tracer Kit (CFDA SE, Thermo Fisher Scientific) according to the manufacturer’s instructions. In brief, cells were washed with 1× PBS and resuspended at 10^6^ cells/mL in working dye solution (1–2.5 µM in 1× PBS) for 15 min at 37 °C. Adherent cells were detached by trypsin before washing and labelling. Five volumes of cell culture medium were then added and cell mixtures were allowed to rest for 5 min to remove free dyes. The labelled cells were centrifuged, resuspended in culture media, and cultured for up to 5 days. The seeding cell number was adjusted to 5 × 10^4^ cells/mL for three different cells so that the plate confluency did not reach 100% at the time of final harvest (Day 5). Labelled cells were harvested Day 1 and Day 5 for flow cytometric analysis using FCS Express 4 Flow Cytometry: Research Edition (De Novo Software, California, USA).

### In vivo tumour model

Animal experiments were approved by the Animal Ethics Committees of SA Pathology and the University of South Australia and conform to the guidelines established by the ‘Australian Code of Practice for the Care and Use of Animals for Scientific Purposes’.

For the orthotopic mouse model of breast cancer, 1 × 10^6^ MDA-MB-231-LM2 cells were mixed with Matrigel (Corning, cat# 354234) (1:1 ratio) and subcutaneously (s.c.) injected as 50 μl into the fourth mammary fat pad of 6–8-week-old female NOD/SCID mice^44^ and harvested before the tumours grew >1 cm^3^.

For pancreatic cancer xenograft model, 2 × 10^6^ AsPC-1 cells were mixed with Matrigel and PBS (Corning, cat# 354234) (1:1 ratio in 30 μl) and injected under the skin in the flank of 6–8-week-old female NOD/SCID/IL-2Rg (NSG) mice and harvested between days 24 and 30 post implantation^46^.

### Immunohistochemistry

Primary mouse tumours were fixed in 10% buffered formalin for 24 h before processing and embedding in paraffin. Sections (4 µm) were cut and subjected to heat-mediated antigen retrieval in pH-6.5 citrate buffer. After cooling for 30 min, the sections were quenched with 1% H_2_O_2_ prior to incubation with anti-CD31 antibody overnight (1:800, Cell Signalling Technology, Danvers, MA, USA), followed by incubation with biotinylated secondary Ab solution (1:500, Abacus dx, Mt Wellington, Auckland, NZ) for 35 min. Sections were then incubated with avidin-biotinylated–horseradish peroxidase complex as per manufacturer’s instructions (Vectastain Elite ABC kit, Vector Laboratories, Burlingame, CA, USA) and visualized using DAB peroxidase substrate solution (ImmPACT™ DAB, Vector Laboratories). Those same sections were further stained using a PAS staining kit from (Sigma-Aldrich, St. Louis, Missouri, USA) according to manufacturer’s instructions before counterstaining with haematoxylin and mounting. Stained sections were scanned by the whole slide image (WSI) scanner (Hamamatsu NanoZoomer Slide scanner). EC-lined blood vessels (CD31+/PAS+) and VM structures (CD31−/PAS+) were further identified within the same tumour section by the presence of RBCs or WBCs in the lumen.

### Detection of human mRNA using quantitative polymerase chain reaction (qPCR)

Quantification of mRNA levels was carried out using qPCR. Total RNA was isolated from excised tumours using TRIzol (Invitrogen, ThermoFisher, Carlsbad, CA, USA, cat# 15596026) and extracted with the RNeasy Mini Kit (Qiagen, Hilden, Germany, cat# 74106). Two micrograms of RNA was reverse-transcribed to cDNA using Superscript III Reverse Transcriptase (Invitrogen, cat# 18080093) with cDNA then subjected to quantitative real-time PCR with QuantiTect SYBR Green PCR kit (Qiagen, cat# 204141). All reactions were performed in triplicate using a Rotor Gene 6000 thermocyclers (Corbett Research, NSW, Australia). Primers were designed for human VE-cadherin (*CDH5)* (F-5′-TGACAATGTCCAAACCCACTCA-3′, R-5′-TGACAACAGCGAGGTGTAAAGAC-3′) and human Ephrin type-A receptor 2 (F-5′-AGACGCTGAAAGCCGGCTAC-3′, R-5′-CAGGGCCCCATTCTCCATG-3′) using Primer Blast (NIH, MD, USA) and purchased from GeneWorks (Thebarton, SA, Aus). Cycling parameters began 15 min at 95 °C, then cycling of 10 s 95 °C, 20 s 55 °C and 30 s 72 °C; for 45 cycles followed by a melt phase. Resultant data were analysed using Rotor-Gene Analysis Software version 6 (Corbett Research). Relative gene expression levels were calculated using standard curves generated by serial dilutions of cDNAs normalised to the human house-keeping gene cyclophillin A (*CycA*) (F-5′-GGCAAATGCTGGACCCAACACAAA-3′, R-5′-CTAGGCATGGGAGGGAACAAGGAA-3′).

### Reporting summary

Further information on research design is available in the Nature Research Reporting Summary linked to this article.

## Supplementary information

Description of Additional Supplementary Files

Supplementary Movie 1

Supplementary Movie 2

Supplementary Movie 3

Reporting Summary

## Data Availability

All data needed to evaluate the conclusions in the paper are present in the paper or in the supplementary materials.
