# Supplementary material for: The development of tumour vascular networks
Source: Commun Biol. 2021 Sep 22;4:1111. doi: 10.1038/s42003-021-02632-x (PMC8458341; doi:10.1038/s42003-021-02632-x)
Supplement: Supplementary file 1 — Description of Additional Supplementary Files [file 42003_2021_2632_MOESM1_ESM.pdf]

Description of Additional Supplementary Files

File name: Supplementary Movie 1.

Description: The formation of vascular networks by endothelial cells. Cells were seeded onto Matrigel and the formation of angiogenesis structures was recorded via microscopy (SV100 Olympus) at 10× magnification over 48 hours.

File name: Supplementary Movie 2.

Description: The formation of vascular networks by breast cancer cells. Cells were seeded onto Matrigel and the formation of vasculogenic mimicry structures was recorded via microscopy (SV100 Olympus) at 10× magnification over 48 hours.

File name: Supplementary Movie 3.

Description: The formation of vascular networks by pancreatic cancer cells. Cells were seeded onto Matrigel and the formation of vasculogenic mimicry structures was recorded via microscopy (SV100 Olympus) at 10× magnification over 48 hours.
