# Supplementary material for: The development of tumour vascular networks
Source: Commun Biol. 2021 Sep 22;4:1111. doi: 10.1038/s42003-021-02632-x (PMC8458341; doi:10.1038/s42003-021-02632-x)
Supplement: Supplementary file 5 — Reporting Summary [file 42003_2021_2632_MOESM5_ESM.pdf]

nature portfolio

Corresponding author(s): Mohsen Dorraki

Last updated by author(s): Aug 11, 2021

Reporting Summary

Nature Portfolio wishes to improve the reproducibility of the work that we publish. This form provides structure for consistency and transparency in reporting. For further information on Nature Portfolio policies, see our Editorial Policies and the Editorial Policy Checklist.

Statistics

For all statistical analyses, confirm that the following items are present in the figure legend, table legend, main text, or Methods section.

n/a	Confirmed	
☐	☒	The exact sample size (n) for each experimental group/condition, given as a discrete number and unit of measurement
☐	☒	A statement on whether measurements were taken from distinct samples or whether the same sample was measured repeatedly
☒	☐	The statistical test(s) used AND whether they are one- or two-sided<br>*Only common tests should be described solely by name; describe more complex techniques in the Methods section.*
☒	☐	A description of all covariates tested
☒	☐	A description of any assumptions or corrections, such as tests of normality and adjustment for multiple comparisons
☒	☐	A full description of the statistical parameters including central tendency (e.g. means) or other basic estimates (e.g. regression coefficient) AND variation (e.g. standard deviation) or associated estimates of uncertainty (e.g. confidence intervals)
☒	☐	For null hypothesis testing, the test statistic (e.g. F, t, r) with confidence intervals, effect sizes, degrees of freedom and P value noted<br>*Give P values as exact values whenever suitable.*
☒	☐	For Bayesian analysis, information on the choice of priors and Markov chain Monte Carlo settings
☒	☐	For hierarchical and complex designs, identification of the appropriate level for tests and full reporting of outcomes
☒	☐	Estimates of effect sizes (e.g. Cohen's d, Pearson's r), indicating how they were calculated

Our web collection on statistics for biologists contains articles on many of the points above.

Software and code

Policy information about availability of computer code

Data collection	We video-recorded the evolution of the vascular networks in a cell incubator under optimised cell culture conditions over a 48 hour period.
Data analysis	The tube-like structures showing VM formation extracted using our custom MATLAB software. The software is explained in Section Methods-The network analysis software.

For manuscripts utilizing custom algorithms or software that are central to the research but not yet described in published literature, software must be made available to editors and reviewers. We strongly encourage code deposition in a community repository (e.g. GitHub). See the Nature Portfolio guidelines for submitting code & software for further information.

Data

Policy information about availability of data

All manuscripts must include a data availability statement. This statement should provide the following information, where applicable:

- Accession codes, unique identifiers, or web links for publicly available datasets
- A description of any restrictions on data availability
- For clinical datasets or third party data, please ensure that the statement adheres to our policy

We video-recorded the evolution of the vascular networks in a cell incubator under optimised cell culture conditions over a 48 hour period.

Field-specific reporting

Please select the one below that is the best fit for your research. If you are not sure, read the appropriate sections before making your selection.

☒ Life sciences ☐ Behavioural & social sciences ☐ Ecological, evolutionary & environmental sciences

For a reference copy of the document with all sections, see nature.com/documents/nr-reporting-summary-flat.pdf

Life sciences study design

All studies must disclose on these points even when the disclosure is negative.

Sample size	Here, 1.5×104 MDA-MB-231 breast cancer cells, 3.5×104 BxPC-3 pancreatic cancer cells and 1.5×104 HUVEC (Human umbilical vein endothelial cells) per well were seeded onto 10 µl of Growth Factor Reduced/normal Matrigel (Corning, Corning, NY, USA) in angiogenesis µ-slides (Ibidi, Munich, Germany) and monitored for up to 48 hrs. Time-lapse video of the tube-formation process in the angiogenesis well was taken on the live cell imaging microscope 'CellVoyager CV1000 Yokogawa Spinning disk Confocal Scanner' (Olympus Life Science, Tokyo, Japan). Images were extracted from the video post-acquisition. We analysed videos captured from breast cancer, pancreatic cancer and endothelial cell types.
Data exclusions	N/A
Replication	The tube-like structures showing VM formation extracted using our custom MATLAB software. Previous studies normally used manual counting approach for measuring tubular structures in VM. This computational approach, based on image processing tools, assists in extracting useful information from VM networks, avoiding miscalculation. Therefore, we developed a software that receives the network images and precisely outputs a number of useful parameters such as number and position of tubular vessels and junctions, histogram, graph parameters, etc.<br>The algorithm consists of the following steps:<br>Step 1) Reading image: Read in the cell images, which are the images of breast cancer cells.<br>Step 2) Image adjusting: The RGB images are converted to grayscale and image intensity values or colormap is adjusted to improve image contrast. In addition, the area of interest is segmented, and any object out of border of area can be removed.<br>Step 3) B/W Filtering: The grayscale images converted to B/W, and after image enhancement, the small holes in vessel images can be removed. Additionally, the scatter points related to a few cells that are not connected to tubular structures may be removed.<br>Step 4) Vessel outline extraction: Using this morphological operation, all objects are reduced to lines in 2-D binary images.<br>Step 5) Finding individual vessel: Tubular junctions are extracted, and the vessels between them identified as an individual vessel. This information assists to calculate the graph parameters.
Randomization	N/A
Blinding	As all experiment has done by computer, blinding was not relevant to our study.

Reporting for specific materials, systems and methods

We require information from authors about some types of materials, experimental systems and methods used in many studies. Here, indicate whether each material, system or method listed is relevant to your study. If you are not sure if a list item applies to your research, read the appropriate section before selecting a response.

Materials & experimental systems

n/a	Involved in the study	
☒	☐	Antibodies
☒	☐	Eukaryotic cell lines
☒	☐	Palaeontology and archaeology
☒	☐	Animals and other organisms
☒	☐	Human research participants
☒	☐	Clinical data
☒	☐	Dual use research of concern

Methods

n/a	Involved in the study	
☒	☐	ChIP-seq
☒	☐	Flow cytometry
☒	☐	MRI-based neuroimaging
